# Hit in the Heart of Life: How Meeting Like-Minded Peers May Contribute to Psychosocial Recovery of Adolescents and Young Adults With Acquired Brain Injury

**DOI:** 10.3389/fneur.2019.00521

**Published:** 2019-05-21

**Authors:** Louise Bakmann, Anne Norup, Birgitte Hysse Forchhammer

**Affiliations:** ^1^Department of Psychology, University of Southern Denmark, Odense, Denmark; ^2^Department of Neurology, Rigshospitalet, Copenhagen University Hospital, Copenhagen, Denmark; ^3^Brain Injury Center BOMI, Roskilde, Denmark; ^4^Traumatic Brain Injury (TBI) Unit, Department of Neurorehabilitation, Rigshospitalet, Copenhagen University Hospital, Copenhagen, Denmark; ^5^The Danish Stroke Association, Taastrup, Denmark

**Keywords:** adolescent, young adult, acquired brain injury, psychosocial, peer support

## Abstract

Adolescents and young adults are often in a particularly vulnerable position following acquired brain injury (ABI). In addition to neurological and cognitive impairment, they are faced with issues concerning education, job, family, and social life. Moreover, they may be limited in meeting peers and may be left alone with psychosocial issues. This paper investigates how this patient group may benefit from meeting like-minded peers. From information gathered through a questionnaire and interviews with participants in a peer support group, the study aimed to investigate the social and psychological advances such a group can offer, and how this may contribute to psychosocial recovery following ABI. Also, the paper indicates how peer support groups may possibly have an impact on the everyday lives of adolescents and young adults with ABI.

## Introduction

According to a Danish study on incidence of acquired brain injury (ABI) in young adults between the age of 15 and 30, a total of 10,542 first-time hospitalizations were identified between 1994 and 2013, making an average of barely 1,200 per year (1). Despite this relatively low number of adolescents and young adults acquiring a brain injury, it is a significant group to consider, as these survivors will probably experience lifelong deficits in many different life areas. Individuals with ABI are usually confronted with a variety of challenges related to physical and cognitive impairments (2–4). It is evident though that the impact of brain injury depends not only upon the type and severity of symptoms, but also upon age at the time of injury onset (5, 6). Young individuals tend to have more unique psychosocial and supportive needs besides specific health concerns, and these issues range beyond physical and cognitive difficulties and include matters related to education, family establishment, relationships, and social activities (5, 7–9). Not only may a sudden and unexpected life event as acquiring brain injury have major implications regarding lifestyle, employment, and social life but additionally, young individuals might have to live with the consequences of injury for most of their lives, often with a dependency on rehabilitation services or instrumental and financial support. Individual concerns and priorities may be different from prior to ABI but furthermore, adolescents and young adults may be confronted with a profound diversion from their anticipated life trajectory (8). The relationship between sudden onset of severe illness and its psychosocial impact on the anticipated life trajectory can be defined by the sociological concept *biographical disruption* (10). By the concept of biographical disruption, it is further suggested that social support can play a significant positive role in adapting to a changed life situation and in regaining a sense of normalization, which is termed *biographical repair* (5, 10).

For adolescents and young adults with ABI, the probability of meeting like-minded peers in hospital settings is low. According to *The Danish Stroke Association*, the number of adults acquiring brain injury in Denmark is about 20,000 (11). Compared to the aforementioned 1,200 patients between 15 and 30 years, there seems to be a domination of older patients and thereby, neurology departments are likely to be dominated by elderly. Consequently, most rehabilitation services may be centered on problems and needs of these patients, and these might be quite distinct from the needs of young patients (6, 12). At the same time, individuals with ABI are often limited in socializing with peers, which could possibly be due to a disruption during education or work, eventually preventing study-related or collegial contacts (13). Moreover, a reduction in socially skilled behavior can be evident particularly following traumatic brain injury (14), and concerns of identity and social norms may arise, inhibiting or even preventing participation in social activities. In helping adolescents and young adults with ABI in obtaining biographical repair, it seems thus necessary to consider psychosocial needs and focus on regaining social abilities to possibly assist these young individuals on their way toward psychosocial recovery.

### Peer Support

Research literature regarding peer support for adolescents and young adults with ABI is limited. However, there is a substantial body of literature indicating effectiveness of using experiential peers in other populations, including psychiatric patients and drug or alcohol addicts [see review by Solomon (15) and Repper and Carter (16)]. Since peer support has proven to be efficient in helping people get through difficult life situations [e.g., (17)], it is found relevant to investigate whether it can be beneficial for adolescents and young adults with ABI. In the literature though, different definitions of peer support are provided, indicating a lack of conceptual consensus. Typically, the term *peer support* defines interventions of social and emotional support offered by people with experience and characteristics similar to recipients (15, 18). Peer supporters are assigned or trained in providing support and can be either financially compensated or volunteers. The overall idea of peer support is letting individuals meet others who have gone through similar life events. Additionally, these like-minded peers can provide advice about strategies based on their own experience, as opposed to advice based only upon theoretical knowledge (17).

Mead and MacNeil (19) have listed some fundamental principles about peer support. These include peer support as not necessarily assuming a specific problem orientation, and as being about mutual responsibility and communication rather than assessment or evaluation, and as focusing on building relationships that support learning and growth. The roles of helper and helpee are not static in that peer support assumes full reciprocity. To supplement these principles, another frequently suggested aspect of peer support is the opportunity to benefit from helping others, traditionally termed *the helper-therapy principle* (20). This principle claims that not only the received help and support is beneficial, but also the act of supporting and helping. In this respect, helping and thereby having an impact on the lives of others may lead to enhanced sense of interpersonal competence and sense of self.

Previous research has suggested that peer support groups can increase social relations and quality of life, which on a societal basis may have economic benefits in the form of reduced number and length of hospitalizations (15). In the present paper, a specific peer support group is presented in which the concept of peer support encompasses the principles defined by Mead and MacNeil (19) and Riessman (20). Additionally, in the group presented here, there is emphasis on like-mindedness, and every participant is as much the supported as the supporting part. Thus, the group is bidirectional and reciprocal, since every participant constitutes a role of a helper *and* a helpee. Despite differences in how far participants have come in recovering from ABI, no one is regarded as more experienced or higher hierarchically placed, and none of them are trained or paid for participating. However, professionals with specialized knowledge about ABI have organized and led group meetings.

### Young Brains—A Unique Peer Support Group

*Young Brains* (*Unge Hjerner*) was conducted as a subproject of the project, *National Study on Young Brain Injury Survivors*, Department of Neurology, Rigshospitalet, Denmark. Young Brains was established in January 2017 with a preliminary duration of 1 year. It was a social intervention with the aim of enabling individuals at the age of 15–30 with ABI to meet like-minded peers. The group constituted a possibility of sharing experience and practicing social behavior, while it had psychoeducational elements by which participants could learn about ABI and related subjects. Patients with affiliation to *The National Study on Young Brain Injury Survivors* in the capital region were invited to join the group. The meetings took place twice a month in a rehabilitation center, and young individuals with ABI were free to participate without specific requirements or commitments.

The concept of Young Brains was not offering therapy *per se* but was rather meant as a supplement to ordinary rehabilitation. For that reason, participants with specific questions related to rehabilitation services, facilities or similar were advised in seeking such through the right channels. A case manager and two neuropsychologists organized and planned meetings, facilitated group discussions, and offered learning opportunities. The content of the meetings alternated between socializing, experience sharing, and presentations by guest speakers with theoretical knowledge related to ABI and youth. Every meeting had a theme and was described in a program sent out for the whole season (themes are listed in Table 1). The meetings always started with an introduction round in which all participants were asked to present themselves by name and age, and they were invited to elaborate as much as they felt like about their brain injury. Also, they were encouraged to raise specific themes for that particular meeting, if they had any in mind. After the introduction round, the participants chose themes they wanted to discuss more thoroughly. Besides group discussions, at some meetings a guest speaker was invited to present a relevant topic about being a young survivor of ABI. At the end of all meetings, participants were asked if they had any questions, and the theme and program of the next meeting was presented.

**Table 1:** Themes of the young brains meetings.

**Meeting**	**Theme**
1. Socializing	
2. Presentation with ABI-related content	Presentation by a medical doctor and a physiotherapist: *Physical training after brain injury: advice, possibilities and limitations*
3. Socializing	
4. Presentation with ABI-related content	Presentation by a well-known Danish neuroresearcher: *The Brain*
5. Socializing	
6. Presentation with ABI-related content	Presentation by a social worker from a job center: *Meeting the local authority*
7. Socializing	
8. Presentation with ABI-related content	Presentation by a neuropsychologist from a rehabilitation center: *Who am I now? – About identity and brain injury*
9. Socializing	Creative activity with an artist (*Unmasking brain injury*)
10. Presentation with ABI-related content	Presentation by an occupational therapist: *Apps for training and daily help*
11. Socializing	Evaluation

### The Present Study

The present study aimed to evaluate and illuminate how a peer support group was received by participants, and how they perceived the effects of participating on their everyday life.

## Methods

### Participants

Participation in the Young Brains group required former or current affiliation to *The National Study on Young Brain Injury Survivors*, a national project in which an age interval of 15 to 30 years was required by the Danish Ministry of Health. Therefore, all participants in this study had been affiliated to the project and at time of the current study, they were between the age of 19 and 32. At the time of data collection, all participants had mild to moderate difficulties related to the ABI. Specific injury related data and data concerning rehabilitation was not collected as a part of the study, as it was conducted in the chronic phase after injury.

### Settings and Procedure

Quantitative and qualitative approaches were combined in the current study, using both questionnaire and interviews. Before producing the questionnaire and the semi-structured interview guide, a psychologist observed the Young Brains group during three meetings to obtain information about the group and the procedure. The study period was from May to mid-June 2017.

A link to an online questionnaire was sent to all participants in the Young Brains group. Five of them were further invited to participate in a semi-structured interview, whereof four agreed on being interviewed. The respondents were selected based on gender, age, participation frequency and time since injury onset. These criteria were set to make sure respondents represented the diversity in the group as much as possible despite the limited number of respondents. The four respondents participated in most of the meetings (half of the meetings or more), so they were familiar with the structure and procedure of the group. The interviews took place one-on-one, either in the out-patient clinic at Rigshospitalet, Glostrup or in the respondents' own homes, and every participant was interviewed once.

#### Questionnaire

The questionnaire of 21 questions was sent out to all participants of Young Brains with a description of the aims of the questionnaire and information regarding how responding was optional and anonymous. The full questionnaire is listed in a translated version in Table 2 and was structured as follows:

Part 1: Demographic information, including gender, age, time since injury, and employment, which enabled examination of pertinent features of the participants.
Part 2: Frequency of participating in meetings and reasons of participating.
Part 3: Participants were asked for their opinion of the different ABI-related presentations.
Part 4: This part was about socializing in the group and consisted of a list of statements, where participants were to select between five levels of agreement. These statements were formulated to explore participants' opinions about Young Brains.
Part 5: Open-ended questions such as what was the best part of Young Brains, if they got anything usable for their everyday life, and what they would change if possible.

**Table 2:** Questions from the questionnaire (translated from Danish).

**Themes**	**Questions**
1. Demographic questions	1. *What is your gender?* – *boy/man* –*girl/woman* 2.*How old are you? _____________* 3.*When did you acquire your brain injury?* –*0–1 years ago* – *1–5 years ago* – *5–10 years ago* – *10 years ago or more* 4.*What is your situation of employment?* – *Studying* – *Working* – *On sick leave* – *Unemployed* – *- Other ______________*
2. About the meetings	5.*How many times have you participated in Young Brains?* – *Every time* – *Most of the times* – *Half of the times* – *A few times* – *I have not participated yet* 6.*Why do you participate in the meetings? (choose one or more)* – *To meet others with ABI* – *To hear the presentations from professionals* – *To get away from home* – *Because others tell me to go* – *To get some advice on how to handle specific problems* – *Because it is nice to be there* – *To tell my own story* – *To hear the stories of others* – *Other ____________* 7.*Please state below how well you think the statements fit the Young Brains group (likert-scale with six degrees of agreement, ranging from “Totally right” to “totally wrong”)* – *A good support* – *A good way of sharing experiences* – *A good way to hear how others handle their situation* – *A chore* – *A break from everyday life* 8.*The program of Young Brains changes from time to time between socializing and ABI-related content. What do you think of this distribution?* – *Not enough socializing and too much ABI-related content* – *Not enough ABI-related content and too much socializing* – *- The distribution is fine*
3. ABI-related content and presentations	9.*State below your degree of agreement in the following statements (likert-scale with six degrees of agreement, ranging from “totally disagree” to “totally agree”)* – *The presentations have been relevant for me (every single theme/presentation was listed)* 10.*Did one of the presentations make a certain impression on you?* 11. *Why did this particular presentation make an impression on you?*
4. Socializing	12.*State below your degree of agreement (likert-scale with 5 degrees of agreement, ranging from “totally disagree” to “totally agree”)*
	– *The participants in young Brains understand me better than my peers* – *I dare to tell about personal worries and problems in Young Brains that I do not tell others about* – *I feel a greater support in the group than elsewhere* – *I get inspired when I hear how other group members handle their problems* – *I get inspired when I hear others in the group tell about their accomplishments* – *By attending to the meetings I have become better at telling about my injury* – *I talk to others in the group about things that I do not talk to others about* 13.*Have you had any contact to other participants in private?* – *Yes* – *No* – *No but I would like to* 14.*Have you had any contact to other participants via SMS, Facebook, or similar?* – *Yes, frequently* – *Yes, one or a few times* – *No* – *- No but I would like to*
	15.*Are you a member of the Facebook group “Young Brains”?* – *Yes* – *No* 16. *How often do you visit the Facebook group?* – *Daily* – *Weekly* – *Monthly* – *Rarer* 17.*What do you use the Facebook group for?* – *To be reminded of meetings* – *To communicate with others in the group* – *- I do not use the Facebook group*
6. Ending and your comments	18.*What do you think is the best part of Young Brains? ___________* 19.*Do you think Young Brains give you anything usable for your everyday life? (please describe below) ____________* 20.*Is there anything about Young Brains that you wish could be different? (please describe below) ____________* 21.*Do you have any ideas about potential future themes, presentations etc.? ____________*

#### Semi-Structured Interviews

A semi-structured open-ended strategy was used in the interviews to allow participants to freely elaborate on their experiences and opinions, which also enabled capturing the unique verbal accounts of the respondents. Initially, they were introduced to the aim and were asked for permission of audio-recording to transcript the responds for later analysis. The duration of the interviews was 30–40 min, and respondents could ask for breaks when needed. The interviews were based on an interview guide, divided into nine sections with distinctive themes, structured as follows:

Part 1: Demographic information was obtained in the same order as in the questionnaire.
Part 2: Structure of Young Brains, including opinions on location and frequency. It also involved questions about the concept of professionals leading discussions.
Part 3: Participants' opinions on the Young Brains program, including the distribution between socializing and learning via professional presentations.
Part 4: Reasons for participating in the group were investigated.
Part 5: Benefits of participating and asked whether anything in the group was not present in other social settings, and if being there was different from being with peers in general.
Part 6: Support and understanding in the group setting compared to personal networks.
Part 7: Sharing experiences.
Part 8: Impact of Young Brains on their everyday lives was investigated, including whether they expected their situation to be different without the group. Also, benefits for everyday life, and changes in their ways of talking about ABI after participation.
Part 9: Young Brains were to be described in three words. In addition to this, participants were asked about the best thing about the group and also, what could possibly be better.

When all questions were answered, participants were encouraged to add points that were not covered throughout the interview. The audio-recordings were transcribed verbatim, and names or potentially identifiable information were anonymized. The transcripts were read multiple times and notes of significant statements were conducted.

## Ethics

The study was conducted in accordance with the Helsinki Declaration. All participants were informed orally and in writing about the purpose of the study, and written consent to participate and to publish data was obtained from all of them. Due to Danish legislation, ethics approval was not required for the present study.

## Results

### Results From the Questionnaire

The questionnaire was completed by 17 participants, including 5 males and 12 females.

#### Participant Characteristics

Participant characteristics are listed in Table 3. The gender distribution was a majority of females (12 out of 17). The age range for the participant group was 19–32 years, making an average of 25.5 years. Out of the 17 participants, 12 were within 5 years of injury onset, and four out of these 12 participants were within 1 year. In relation to employment, eight were studying and one was employed at the time of the study (not further specified). The remaining eight participants were unemployed, early retired, or on sick leave. Regarding frequency of participation in the Young Brains meetings, 13 had participated in half of the meetings or more, while the remaining four had participated in less than that.

**Table 3:** Characteristics of the participants.

**Gender**	**Age**	**Time since injury**	**Employment at the time of the study**	**Participation in meetings**
Female 76% (12)	19–21 29.5% (5)	<1 year 23.5% (4)	Studying 47% (8)	All times 11.5% (2)
Male 24% (5)	22–24 17.5% (3)	1–5 years 53% (9)	Employed 6% (1)	Almost all times 47% (8)
	25–27 17.5% (3)	6–10 years 11.5% (2)	Unemployed, early retired, on sick leave 47% (8)	Half of the times 17.5% (3)
	28–32 35% (6)	>10 years 11.5% (2)		A few times 17.5% (3)

#### Reasons for Participating in Young Brains

Participants were asked to choose one or more reasons of participating in Young Brains among eight options. As is seen in Table 4, all participants chose the option *To meet other young individuals with ABI*. Other reasons commonly selected were *To get advice on how to handle specific problems* (15) and *To listen to professional presentations* (15). No one stated that others told them to go.

**Table 4:** Reasons for participating in young Brains.

**Responding options**	**Percentage of times chosen (%)**
To meet other young individuals with ABI	100
To get advice on how to handle specific problems	88
To listen to the professional presentations	88
Because I like the atmosphere	76.5
I want to hear the stories of others	65
It feels good to tell my own story	47
To get away from home	18
Others tell me to go	0.00

#### Participant Benefits

To investigate benefits of Young Brains, participants were asked to rate their degree of agreement on eight statements. The highest level of agreement was on statements describing Young Brains as *A great support, A good way of sharing experience*, and *A good way of hearing how others handle their situation*. No one responded with the rating of Young Brains as being *A chore*. Other results reveal that most of them felt more understood and supported by peers in the group compared to other social networks. The majority of participants (13 out of 17) stated that they could talk about personal concerns and problems in the group that they did not tell others, and 13 also replied that participation made it easier to talk about their brain injury elsewhere. When asked if Young Brains had had any benefits in everyday life, participants replied in a free text box. One mentioned courage and perspective: “*It gives me courage to move on and continue fighting in my everyday life. It helps me to see things from another perspective […]”* (m, 21). Another highlighted understanding: “*I get help dealing with challenges that weigh heavily on my shoulders and are hard to explain to others. With them I don't have to explain for 15 minutes – the understanding is there right away. That's a relief; it calms me down.”* (f, 26). Others wrote how they had become more open and accepting about the consequences of their injury.

### Results From the Interview

#### Participant Characteristics

Four participants (*m* = 2, *f* = 2) were interviewed. Since completing the questionnaire was anonymous and sent to all participants in Young Brains, respondents might possibly have participated in both the questionnaire and the interview. Characteristics of the respondents at the time of the interview are listed in Table 5.

**Table 5:** Respondent characteristics.

**Respondent**	**Gender**	**Age**	**Employment**
R1	Male	21	Studying
R2	Female	32	Employed (part time)
R3	Male	23	Employed (full time)
R4	Female	31	Studying

#### Reasons for Participating in Young Brains

The respondents were asked why they initiated participating in Young Brains, and why they continued to participate. All respondents stated the main reason as a need of meeting like-minded peers as both the reason of initiating and continuing participation. One respondent further elaborated “*It originated in my need of finding a place to fit it”* (R4).

#### Themes

In analyzing the interviews, three principal themes were identified, each with three corresponding subthemes as illustrated in Table 6.

**Table 6:** Themes and subthemes identified in the interviews.

**Core theme**	**Subthemes**
Socializing	A supportive network Mutual and reciprocal understanding A place to belong
New knowledge	Motivation, inspiration and collective problem-solving Professional guidance Practicing skills in a safe environment
Psychosocial well-being	Relief of concerns Accept and normalization Sense of purpose and social value

#### Socializing

##### A Supportive Network

Most respondents stated having great supportive networks, but did not feel truly supported or understood by them. One respondent uttered: “*I think family and friends support me as well as they can, but when the real understanding isn't there, it's difficult”* (R2). In general, respondents had difficulty in talking to friends and family about the injury, which often amplified feelings of loneliness and disconnecting from emotional and social support. Some avoided talking about their injury and did not want to bother their relatives who were already personally and emotionally involved. As a result, respondents had rarely spoken about injury-related concerns, and most of them had not openly expressed their worries and frustrations before participating in Young Brains.

All respondents stated profound differences on the support provided in Young Brains compared to other networks. “*They understand what's difficult and what the closest friends don't understand […]. Well, my friends try but I can feel it's not the same.”* (R2). Respondents described Young Brains as a supplement to family and friends, and all stated that the group made them feel more supported and understood than in other networks. They described Young Brains as offering more authentic in-depth feelings of support, and because acknowledgment and feedback was given from like-minded peers, they felt a higher and more significant value of the support.

##### Mutual and Reciprocal Understanding

Respondents expressed difficulty in relating to even close relatives following ABI. They felt challenged by the invisibility of their injury and often had to explicate how they could not do the same things to the same extent as before, as in the following example: “*I cannot stay out until 3:AM anymore and they don't really understand. And they often put pressure on me, right? They say “Are you leaving early again?” and things like that.”* (R3). Most respondents had even experienced people doubting on the consequences of ABI, and some mentioned this as both impeding participating in social activities and as changing their social relationships. Two respondents explained how they did not perceive their relationships with friends and family as reciprocal anymore, and one of them opposed this to his experience in Young Brains: “*In Young Brains we are all equal. No matter what our background is. It's our illness that ties us together. I don't consider anyone better than others* […]*. We're all equal”* (R1). He felt a connection due to reciprocity and continued: “*On the outside I look like a completely normal guy and nothing is wrong with me. But everyone in Young Brains knows. It's on the inside something's wrong.”* (R1).

In general, respondents could relate and understand their peers in Young Brains, and when sharing individual accomplishments, they felt like overcoming challenges together. One respondent explained her way of relating to the victories of others: “*It's nice to go somewhere where people understand the upturn swell of reading 40 pages in a row or something. Maybe they don't understand exactly about the 40 pages, but they can hear the way I'm telling it and they can translate it to their own injury. Another participant once said “I've cut over an avocado on my own.” […] You can feel it in the way she tells it – Hey, that's like when I read my book!”* (R4).

##### A Place to Belong

None of the respondents had previously encountered peers in similar situations. Confronted with completely changed life situations, it was hard to find their place when being with others, and through observation at group discussions it became clear, how the participants faced new challenges and contrasts to the lives they lived before. For all respondents in the study, Young Brains constituted the first meeting with like-minded peers, which made them discover that they were not alone in being a young survivor of ABI. Participants found a place to fit in and be accepted both despite and in virtue of being young with ABI.

#### New Knowledge

##### Motivation, Inspiration, and Collective Problem-Solving

Respondents felt motivated by each other. By listening to how peers handled individual challenges, they felt encouraged to try something alike to handle theirs. Some participants stated that telling about accomplishments increased their self-confidence and enhanced their motivation. They found it inspiring, motivating and instructive to hear about the challenges of others, and some even saw individual accomplishments as common victories: “*I think we all get happy when hearing about the success or fight of another person. In a way we have been on the sidelines and seen the fight and then… Who wouldn't be happy when a person comes back and tells you that he won that fight?”* (R1). Thus, it was not only inspiring to hear about specific problems but also to just listen to peers telling it was possible to overcome struggles.

Related to sharing experience, one respondent expressed the following: “*Maybe you come up with five tools of handling your problems, but after two hours in the group, you'll have five or six new”* (R1). Another had a similar experience of seeking advice in the group: “*When you say “I find it hard to settle down and I can't sleep” or something, they give you like eight options of what to do[…], and there are no hard feelings about using the advice or not.”* (R4). The group was used as a way of gaining motivation and courage to face challenges, and not least as a forum of collective problem solving where specific problems and potential solutions could be discussed.

##### Professional Guidance

All respondents in the study expressed an importance in professionals organizing the meetings. One stated reason for this was that it enabled a more objective viewpoint as a supplement to participants' subjective experiences. However, it cannot be concluded whether participants would have a similar experience with a different group design or in another setting. More than focusing on neurological and physical impairments, the professionals of Young Brains had a focus on both brain processes and potential consequences of this however, they also emphasized a focus on issues concerning adolescence and young adulthood.

##### Practicing Skills in a Safe Environment

The interviews reveal that new knowledge was not only gained through listening to peers and professionals. Participants used the group to practice social and communicative skills, and when asked if participation had any impact on their way of talking about their injury, they all replied that it was easier to talk about their injury after discussing it in the group. By talking freely about it there, they did not make a big deal of talking about it in general. Some participants were convinced that discussions in Young Brains made them more open about their situation and more able to accept it. They practiced how to put their own situation into words and to talk about it without being emotionally overwhelmed. One respondent described how sometimes she felt even too open and straightforward: “*Sometimes I get into situations, where it's easy to say “Well, that's just because I had two strokes,” and people are like “Excuse me, what did you say? Aren't you dead then?”, but anyway I think it's a relief to have come so far that I can tell it in that way.”* (R4).

#### Psychosocial Well-Being

##### Relief of Concerns

One respondent was uncomfortable about talking to relatives about his injury, but he had no problem of mentioning it in Young Brains, where he could freely express worries and thoughts of guilt or frustration. Another one explained how she had removed injury-related concerns from everyday life by letting it out at the meetings. She used Young Brains as a kind of parking lot for issues related to the injury, with the result of her everyday life not being overshadowed by negative thoughts and worries. In Young Brains she could place more and more of her injury-related thoughts, thereby diminishing it from other settings and relations. In the interview, she elaborated her need of having a place where *it* could be, with *it* referring to her injury: “*For me it's about having somewhere, where* ***it****has a place […]. That part of me filled everything once and had no place to be. That part of me is no longer that big, but that part of me – I still need it to have a place somewhere. I don't want it to fill my whole everyday life, and that's why it's nice to have somewhere to go, where* ***it****can be”* (R4).

##### Accept and Normalization

Respondents described their lives as drastically changed after their injury. They were challenged by impairments and had concerns related to the question: Who am I now? One respondent described a need of peers to share experiences with, thereby being validated, as this anecdote about delivering an examination paper illustrates: “*I felt sick for like one and a half weeks afterwards. I couldn't understand what happened, and then we got to talk about energy management and fatigue and about spending energy […]. When we talked about it, I understood, and it made sense, and then I didn't understand how I couldn't have understood. One is just firmly anchored in a “before I got ill”-understanding of oneself […].”* (R4). By talking to peers in Young Brains, she gained a comprehensive explanation of why she felt as she did and could accept it better. She further highlighted that meeting like-minded peers gave her a real and credible base of comparison: “*It's important for me to have a perspective. If I were to compare myself with people that do not have a damage to the brain, I'm just bad at everything”* (R4). Others also mentioned the opportunity of viewing their situation from new perspectives. They met peers with similar or even more challenging issues, which made them reflect on their own situation, though some also felt ambivalence when listening to peers with more significant or visible impairments. Comparing oneself with others generally had a positive impact and constituted a way of challenging beliefs about ABI. Further, it contributed to a sense of normalization and a greater acceptance of their situation.

##### Sense of Purpose and Social Value

An important benefit gained from Young Brains was a feeling of helping others. By sharing personal stories, successes and challenges, respondents inspired each other to face challenges, which contributed to a sense of empowerment. When asked if they felt like helping others, all replied that they did not do anything special, though most of them had been told that something they said was helpful. Helping others contributed to convictions that their experiences were helpful for others. According to this, one respondent said: “*In a way it's about having experiences that have costed so much, right? […] In a way you need it to help somewhere in the world, right?”* (R4).

## Discussion

The aim of the study was to evaluate and illuminate how a peer support group was received by a group of young ABI survivors, and how they perceived the effects of participating on their everyday life. There was no data collection before participating in the group why it cannot be concluded whether the group *per se* made a difference for participants. However, based on the results, tentative conclusions are discussed in terms of how peer support groups could possibly contribute to fulfilling psychosocial needs and thereby assist adolescents and young adults with ABI on their way toward psychosocial recovery.

### Hit in the Heart of Life

When acquiring a brain injury during adolescence or young adulthood, the individual is figuratively speaking “hit in the heart of life,” meaning drastic disruptions and significant deviations from their anticipated life trajectory, and in a time where they are usually not yet settled, when it comes to family, education, career, etc. Peer support has proved valuable in many populations, and the current study indicates how it may also be beneficial for young ABI survivors. Being young with ABI may induce a variety of concerns and challenges related to finding oneself in a drastically changed life situation, but they are often left alone to fulfill psychosocial and supportive needs. Prior research has claimed that peer support can enhance social networks and increase quality of life [see review: (15)] and is suggested to fulfill emotional and social needs after unexpected neurological events (21, 22). This seems consistent with the findings in this study.

### Are Peer Support Groups More Beneficial Than Other Social Networks?

Adolescents and young adults with ABI rarely meet patients of their own age. For most participants in this study, Young Brains was their first meeting with peers with ABI, and the results of the questionnaire and interviews equivocally reveal the main reason of participating in the group was meeting like-minded peers. Having a place to express worries and thoughts and have it acknowledged by peers fostered feelings of not being alone. Moreover, receiving feedback and understanding from peers seemed to make participants feel validated on their experiences and difficulties. Participants in the group felt understood and supported in ways they did not feel elsewhere, even if they had supportive social networks. Based on the interviews supplied with results from the questionnaire, it is revealed that participants felt free to talk about personal issues, and they did not have to explain or defend themselves, possibly because they were met by reciprocal and like-minded peers. Thus, according to the results, peer support groups can possibly have beneficial psychosocial elements for adolescents and young adults with ABI. This is consistent with prior research on peer support groups showing positive outcomes on various psychosocial constructs, including self-confidence, adaptation to disability etc. (5, 23, 24).

### Can Peer Support Groups Reduce Social Isolation?

Social isolation is a frequent consequence of ABI. Previous research suggests that peer support groups can play a role as a social gathering and replace limited or even lost social opportunities (5), which seems to be confirmed in this study. Participants in this study could test their own limits and which considerations to take in social settings. Thereby the group constituted a safe environment to develop social and communicative abilities without being judged or stigmatized. The group did not require much more from participants than showing up, and respondents from the interviews emphasized a clear understanding of fatigue, lack of resources, etc. Consequently, participating in the group was considered participating in a social activity on their own terms. This clearly played a role in how participants talked about themselves and their brain injury in general. By telling their stories and sharing challenges and accomplishments in the group, not only did their relation to each other grow, but they also practiced how to express themselves and communicate with others. Accordingly, the group might have contributed to development of social abilities and thereby constituted a way of reducing the risk of social isolation.

### How Can Participants Benefit From Each Other?

Participants expressed how they motivated and inspired each other and benefitted from the experiences of others. In the questionnaire, it was stated specifically that they participated to hear others tell their story rather than to tell and share their own story. Respondents from the interviews claimed to be introduced to new ways of thinking and increased courage to face challenges, but the group also contributed to a sense of meaning and value when sharing experiential advice, consistent with the helper-therapy principle (20). Participants felt like gaining a sense of empowerment and social value since they were contributing to the recovery and well-being of others. Furthermore, personal experiential knowledge made it possible to use the group for collective problem solving in the sense that participants raised questions about specific issues, and peers made suggestions on how to deal with it. Thus, problems were collectively discussed, and concurrently the successes of individuals were perceived as common victories. However, the study did not measure whether these experienced benefits reflect real changes in the lives of the participants, but only their subjective descriptions of their experiences of participating in the group.

### Does Participation in a Peer Support Group Have an Impact on Everyday Life?

In investigating whether peer support groups can contribute to psychosocial recovery, an important question is whether participation affected the everyday lives of participants. Based on the results, the answer to this question is clearly positive. Our method do not allow us to conclude, but the results from the questionnaire reveal high degrees of agreement on statements saying that they learned new strategies of dealing with ABI, they found a place to fit in and felt understood and supported. The interviews further revealed that respondents became part of a reciprocal network, made friendships, and gained a sense of social value. However, a very important profit of the group was an increased accept of their situation. By meeting like-minded peers, they got a reliable basis of comparison; they got to see themselves from new perspectives and were challenged on their beliefs. Moreover, the group served as a place to put injury-related issues, thereby eliminating it from everyday life. Whereas, support groups may be perceived as beneficial, it is not yet proven that they do provide a step toward well-being in everyday life.

### How Can Peer Support Contribute to Psychosocial Recovery?

Respondents from the interviews reported that they often felt alone and avoided talking to relatives about injury-related concerns. They found it hard to relate to peers on one side and to ABI patients on the other, since many of them had not met any patients of their age before. Participants felt a special connection to each other and felt understood and supported in distinct ways, compared to other networks, which was also confirmed by the results of the questionnaire. Also, participants were inspired and motivated to try out new strategies of handling challenges, and they practiced social and communicative abilities. They learned to view themselves from new perspectives and got reliable basis of comparison. More of them stated that they had become more open about their injury and got to accept their situation. Moreover, a part of the concept of Young Brains was to provide psycho-educative features. By listening and asking, participants clearly gained knowledge about ABI and youth. Thus, following an ABI-induced biographical disruption, a peer support group may contribute to biographical repair and thereby to some degree of psychosocial recovery.

## Clinical Implications and Recommendations

Though this study indicates that peer support groups might be beneficial for psychosocial recovery following ABI, young ABI survivors with behavioral difficulties could potentially have inhibitory impact on group discussions. Further, it may not be as beneficial in the acute phases of injury, since participating may be quite demanding for newly injured individuals due to medical matters, fatigue, existential crisis, etc.

The group was found to be highly dependent on professionals with experience from ABI treatment to organize and lead meetings. Their role was not only planning but also to form a frame and frequently explicate this, which included facilitating participation with specific focus on guiding and supporting participants with behavioral or other barriers for participation, related to their ABI. It was further revealed that participants had a need of contact to the professionals between meetings to ask clarifying questions.

When it comes to considerations on location, some individuals with ABI have physical difficulties why location for group meetings may include availability for wheelchairs and people with impaired mobility. Also, participants in this study stressed the importance of easy access by car and public transportation.

### Limitations in This Study

Originally, the data collection of this study was completed in order to evaluate Young Brains as a social intervention. Therefore, the questionnaire and the interviews were centered on questions on the intervention *per se*, and on the yields of this specific group design. This means that there is no data collected from before participating in the group and thereby no such data for comparison. Moreover, detailed participant characteristics were not collected as part of the not addressed in this study.

The results of this study are based on the experiences of a small group of young ABI survivors (*n* = 4 in the interview and *n* = 17 in the questionnaire). These participants actively participated in the intervention for several months and thus, results are based on experiences and reflections from participants, who were positively minded and who generally found the intervention meaningful and beneficial. Therefore, one has to be careful to generalize findings, and there is still a need of research focusing on the characteristics of young ABI survivors, who might benefit from this kind of intervention.

## Conclusion

Peer support groups might play a significant role in assisting adolescents and young adults with mild to moderate ABI toward psychosocial recovery. Young ABI survivors are often drastically disrupted in an age and life stage that is already quite unsettled and demanding, in other words hit in the heart of life. This study reveals insight in how meeting like-minded peers may be beneficial by enhancing psychosocial adjustment of adolescents and young adults with ABI. Thus, age-appropriate peer support groups could possibly fulfill a special role not usually met in the structure of rehabilitation services, though more research is needed on this topic. Participants in this study experienced they could provide comprehensive understanding and support to each other that was not found elsewhere. However, there is also still a need for research that provides more knowledge as to what characterizes patient groups, who could profit from this kind of intervention. Furthermore, we lack knowledge of whether peer support groups actually influence other parameters than self-perceived outcome.

## Ethics Statement

The study was conducted in accordance with the Helsinki Declaration. All participants gave written consent to participate.

## Author Contributions

AN, BF, and LB contributed conception and design of the study. LB formulated the questionnaire and the interview guide, collected and analyzed data. LB wrote the first draft of the manuscript. AN and BF wrote sections of the manuscripts. All authors contributed to manuscript revision, read and approved the submitted version. The corresponding author takes primary responsibility for communication with the journal and editorial office during the submission process, throughout peer review and during publication. The same author is also responsible for ensuring that the submission adheres including details of authorship, study ethics and ethics approval, etc. The corresponding author should also be available post-publication to respond to any queries or critiques.

### Conflict of Interest Statement

The authors declare that the research was conducted in the absence of any commercial or financial relationships that could be construed as a potential conflict of interest.
